# Desconexão de cateter para quimioterapia: uma complicação rara?

**DOI:** 10.1590/1677-5449.007116

**Published:** 2016

**Authors:** Alexandre Faraco de Oliveira, Horácio de Oliveira

**Affiliations:** 1 Universidade do Planalto Catarinense – UNIPLAC, Lages, SC, Brasil.; 2 Clínica Ana Carolina, Lages, SC, Brasil.

**Keywords:** cateteres venosos centrais, falha de equipamento, dispositivo de acesso vascular

## Abstract

A utilização de cateteres totalmente implantáveis no tratamento quimioterápico constitui uma necessidade que acarreta o risco de múltiplas complicações, algumas inerentes à inserção do dispositivo e outras relacionadas ao próprio cateter. Relatamos três casos nos quais o cateter apresentou-se desacoplado de seu respectivo reservatório. No primeiro caso, ocorreu a desconexão do cateter de seu respectivo reservatório, e nos outros dois casos, verificou-se a fragmentação do cateter. Em todos os casos, foi necessária a retirada endovascular do cateter. Tal desfecho é apontado como raro, mas costuma estar presente na maioria das revisões e traz consigo o risco de complicações graves, ainda que frequentemente seja assintomático. É desejável o acompanhamento de pacientes que possuem tais cateteres a fim de que se possa detectar precocemente tais complicações e compreender os fatores que determinam o aparecimento dessas situações.

## INTRODUÇÃO

Os cateteres utilizados para infusão de quimioterápicos são um instrumento bastante útil e, por vezes, fundamental para a realização do tratamento oncológico. Seja devido à ação irritante da droga nas veias dos membros superiores ou à necessidade de múltiplas sessões, a ausência de um acesso adequado impossibilita o tratamento em diversos casos^1^.

O cateter totalmente implantável (CTI) do tipo *port-a-cath* tende a ser a escolha principal, pois, uma vez instalado, permite o acesso permanente a uma veia profunda, necessitando apenas a punção de seu reservatório para tal. Oferece, além da infusão de medicamentos, a possibilidade de coleta de sangue para exames. Com a ampliação dos tratamentos quimioterápicos existentes e a sobrevida cada vez maior dos pacientes proporcionada por esses tratamentos, tais cateteres passaram a ser utilizados em maior quantidade e por maior tempo^1^.

Existem diversas complicações associadas tanto à implantação quanto ao uso de tais dispositivos. As complicações mais graves são relacionadas ao implante propriamente dito, como pneumotórax ou hemotórax, e costumam manifestar-se imediatamente, sendo relacionadas à escolha do local de punção. Complicações mais comuns, como hematoma ou infecção no local da punção, tendem a ocorrer mais tardiamente, mas costumam gerar poucos riscos ao paciente e ser facilmente detectadas^2^.

Neste trabalho, relatamos três casos nos quais o cateter apresentou-se desconectado de seu reservatório sem que houvesse causa aparente para tal. Ele se tornou um corpo estranho no sistema venoso profundo, especificamente na cava superior, acarretando o risco de complicações potencialmente graves e de difícil diagnóstico. A seguir, faremos uma revisão para situar tais complicações em relação aos relatos existentes.

## RELATOS DOS CASOS

**Figura 1:**
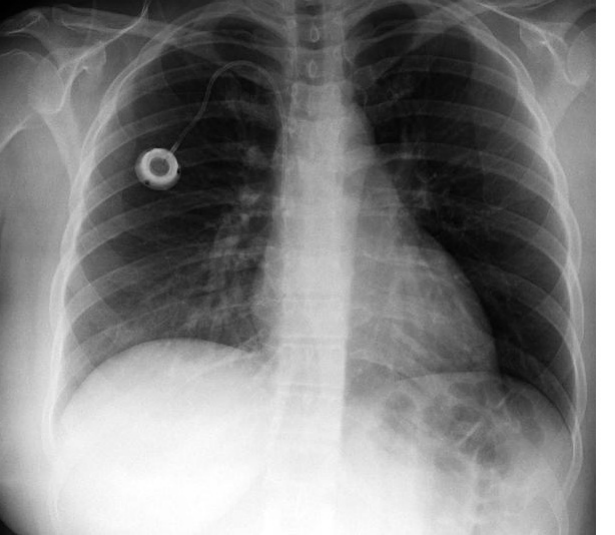
Radiografia de tórax demonstrando reservatório e cateter implantados através de punção em veia subclávia direita.

**Figura 2:**
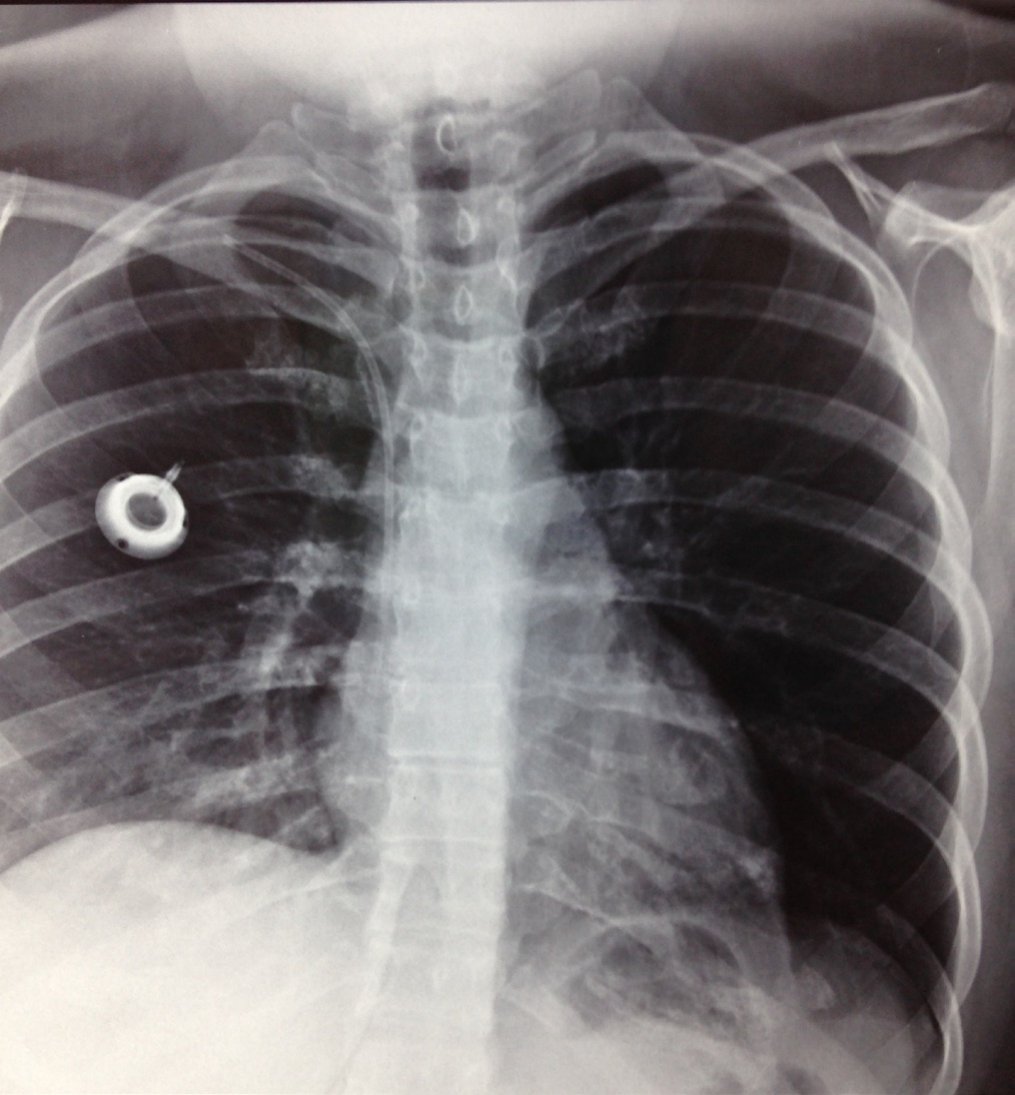
Cateter desconectado de reservatório implantado em tórax à direita.

Caso 1: paciente feminina, 28 anos, apresentou neoplasia de mama esquerda durante a primeira gestação, sendo submetida a mastectomia esquerda com aproximadamente 26 semanas de gestação. Com 34 semanas de gestação, foi realizado o parto cesariano, seguido do implante de cateter para quimioterapia através de punção em veia subclávia direita e fixação do reservatório no tórax (Figura 1). Cerca de 10 dias após o implante do cateter, a paciente queixou dor cervical à direita associada à tumefação em trajeto de veia jugular interna direita e dor à palpação local. Foi realizada nova radiografia de tórax, que evidenciou desconexão entre o cateter e o reservatório (Figura 2). Foi então realizada ultrassonografia com Doppler cervical, que evidenciou trombose da veia jugular interna. A paciente foi submetida a anticoagulação, apresentando regressão completa dos sintomas relacionados à trombose após 15 dias. A anticoagulação foi suspensa por 72 horas. Em seguida, a paciente foi submetida a retirada endovascular do cateter através de cateterização via veia femoral comum direita, com posterior reintrodução da anticoagulação.

**Figura 3:**
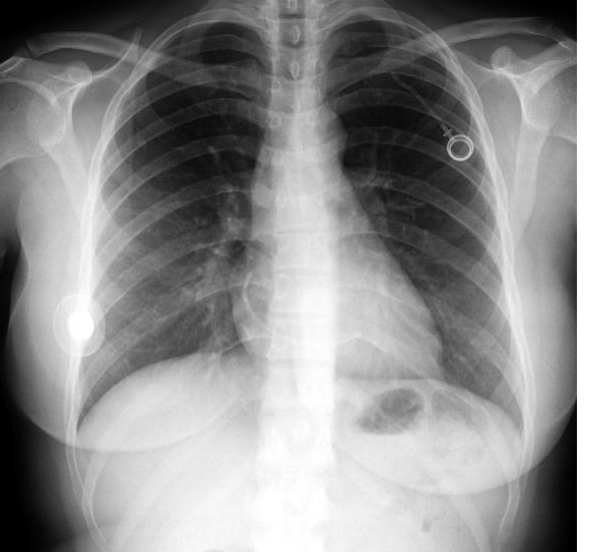
Radiografia demonstrando o reservatório e parte do cateter implantados em tórax à esquerda, com maior porção do cateter embolizada na área cardíaca.

Caso 2: paciente feminina, 33 anos, apresentou neoplasia de mama direita, sendo submetida a mastectomia direita. Aproximadamente 90 dias após a cirurgia de mama, foi submetida a implante de cateter para quimioterapia a partir de punção em veia subclávia esquerda, com fixação do reservatório em tórax à esquerda. A paciente utilizou o acesso para a realização de cinco sessões de quimioterapia em um período de 5 meses, não havendo relatos de qualquer dificuldade de utilização do acesso. Ao comparecer para a sexta sessão do tratamento, o cateter não demonstrou refluxo ao ser puncionado, e a paciente foi encaminhada ao serviço vascular. Realizada radiografia de tórax, evidenciou-se o reservatório e um pequeno segmento do cateter implantados em tórax à esquerda, e um pedaço de cerca de 10 cm do cateter alojado na projeção da área cardíaca (Figuras 3 [Fig gf04]-5). A paciente apresentou-se assintomática, negando dor ou desconforto torácico, taquicardia ou taquipneia. A paciente foi então submetida a retirada endovascular do cateter através de cateterização via veia femoral comum direita.

**Figura 4 gf04:**
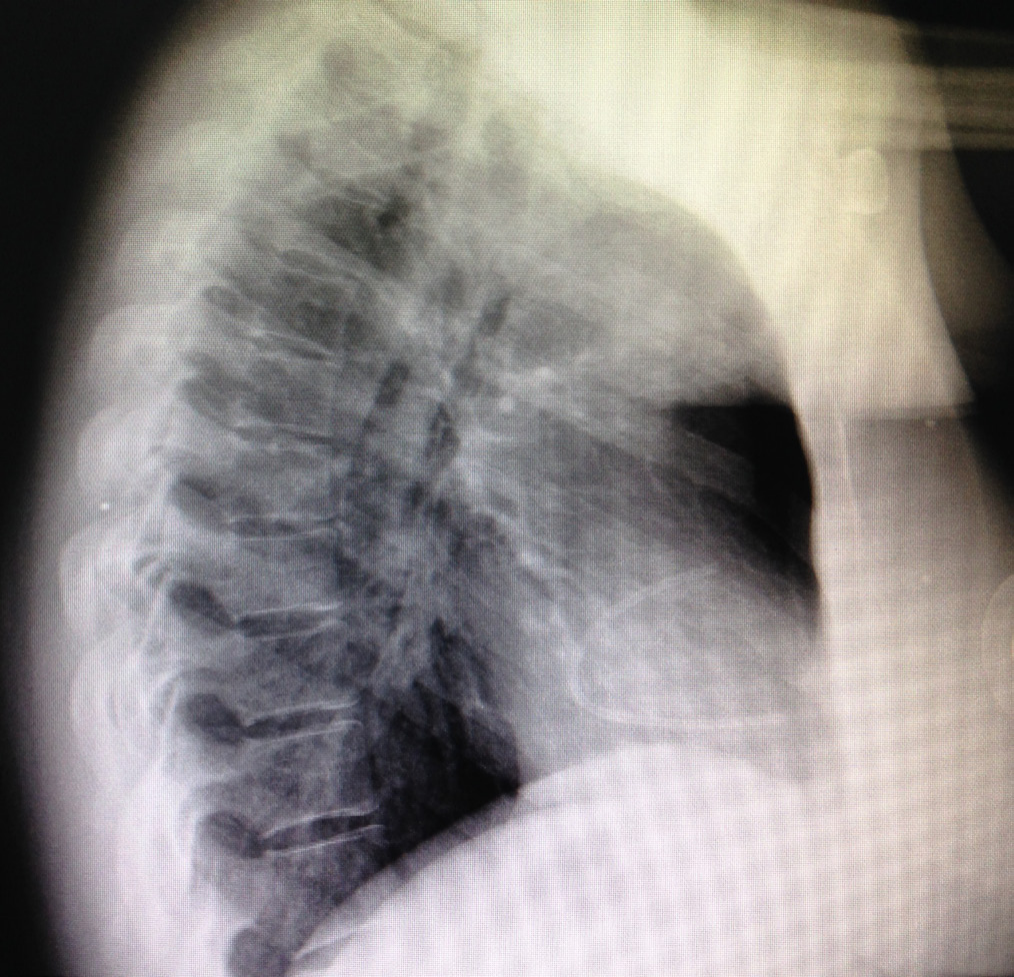
Radiografia demonstrando o reservatório e parte do cateter implantados em tórax à esquerda, com maior porção do cateter embolizada na área cardíaca.

**Figura 5:**
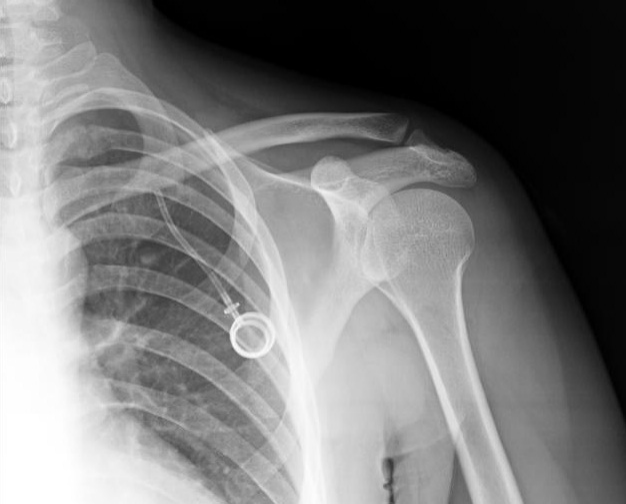
Radiografia demonstrando o reservatório e parte do cateter implantados em tórax à esquerda.

**Figura 6:**
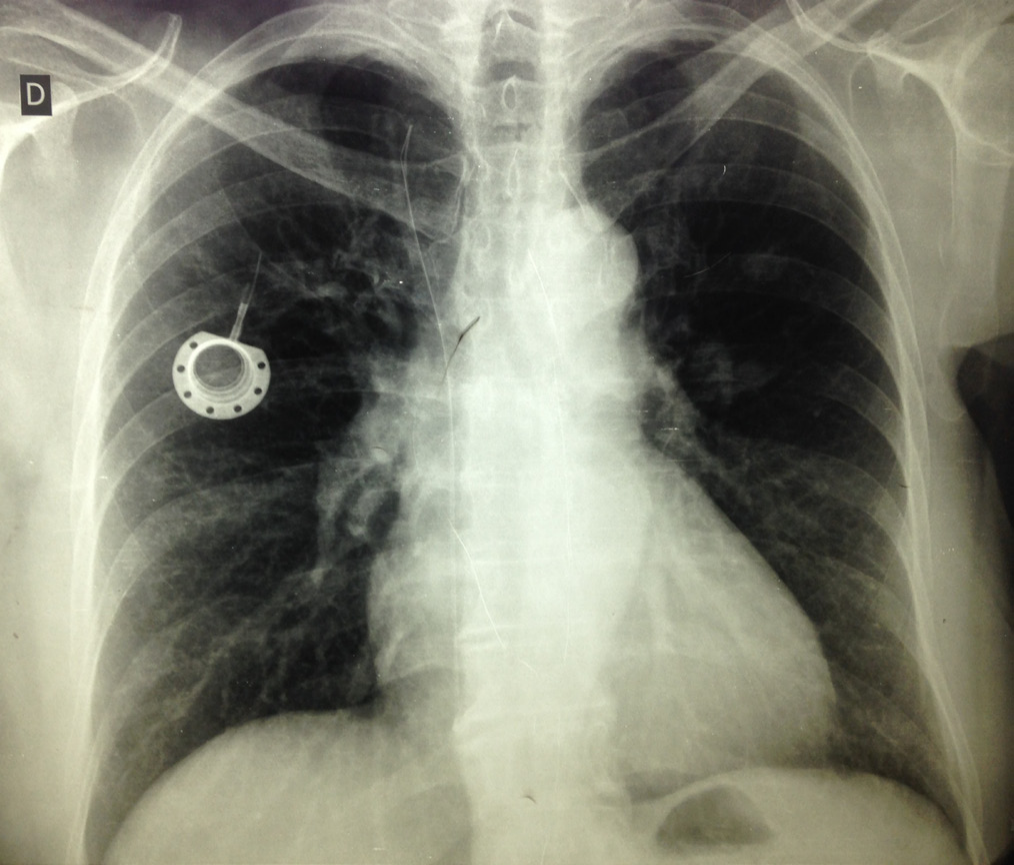
Radiografia de tórax demonstrando cateter fragmentado e reservatório implantado em tórax à direita.

Caso 3: paciente feminina, 60 anos, apresentou neoplasia de mama direita, sendo submetida a mastectomia direita. Aproximadamente 60 dias após a cirurgia de mama, foi submetida a implante de cateter para quimioterapia a partir de punção em veia subclávia direita, com fixação do reservatório em tórax à direita. A paciente utilizou o acesso para a realização de múltiplas sessões de quimioterapia, em um período de 19 meses, não havendo relatos de qualquer dificuldade de utilização do acesso. Ao comparecer à 20ª sessão do tratamento, o cateter não demonstrou refluxo ao ser puncionado, e a paciente foi encaminhada ao serviço vascular. Realizada radiografia de tórax, observou-se a fragmentação do cateter (Figura 6). A paciente apresentou-se assintomática, negando dor ou desconforto torácico, taquicardia ou taquipneia, sendo, em seguida, encaminhada para a retirada endovascular do cateter.

## DISCUSSÃO

A implantação de cateteres para realização de tratamento quimioterápico é um procedimento já bastante conhecido e com ampla utilização, de forma que as complicações mais frequentes também são bastante conhecidas^1^.

Existem complicações diretamente relacionadas ao local de punção e implantação do dispositivo. Embora existam diversas possibilidades para implante de tais cateteres, três acessos costumam ser os mais utilizados: a punção de veia subclávia com fixação do reservatório no tórax, a punção ou dissecção de veia jugular com fixação do reservatório no tórax e a dissecção de uma veia de membro superior, cefálica ou basílica, com fixação do reservatório nesse mesmo membro superior. Em todos os casos, busca-se situar a extremidade distal do cateter na cava superior, bem como posicionar o reservatório de forma a facilitar sua punção local^3^.

As complicações mais comumente relatadas, hematoma e infecção do local cirúrgico, são comuns a todos os acessos. A inserção do cateter através de dissecção, seja em veia cervical ou em membro superior, costuma estar relacionada a um tempo cirúrgico maior, à dissecção de uma área mais ampla e, eventualmente, à necessidade de sedação associada a anestesia local^2^^,^ 4.

O acesso através da punção de veia subclávia acarreta riscos específicos a punção de veias profundas do tórax, ou seja, o pneumotórax e o hemotórax, bem como a punção inadvertida de artérias. Entretanto, costuma ser um acesso menos traumático para o paciente, que permite a fixação do reservatório na parte superior do tórax em local favorável para a punção. Seja qual for o local do acesso, a trombose associada ao cateter também é uma complicação presente, ainda que pareça estar mais relacionada ao acesso realizado por dissecção nos membros superiores^2^^,^ 4 ^,^ 5.

O risco inerente à punção relacionado ao acesso através da veia subclávia tende a ser minorado pela experiência do cirurgião, bem como pela utilização da ultrassonografia como guia^6^. Ademais, os problemas relacionados à punção propriamente dita tendem a se manifestar de forma imediata, sendo facilmente identificados^4^.

Entretanto, o acesso por punção da veia subclávia carregará permanentemente o risco de uma complicação específica, denominada síndrome de Pinch-off, que consiste no pinçamento do cateter entre a clavícula e a primeira costela, com consequente fratura parcial ou total do cateter^7^^-^ 9. A revisão sistemática de cateteres implantados em veia subclávia demonstrou que a lesão do cateter com microrrupturas pode ser mais comum do que se supunha e pode estar relacionada ao tipo de material utilizado^10^.

Embora os CTIs apresentem um formato semelhante, existem diferenças pontuais na constituição desses materiais. Os reservatórios podem ser de material plástico ou metálico, e os cateteres, de silicone ou poliuretano. Existem evidências de que os cateteres de poliuretano são mais propensos a complicações trombóticas e infecciosas, enquanto os de silicone são mais sensíveis a eventos mecânicos como desconexão e ruptura^10^.

Os casos relatados tratam de duas situações que têm como resultado comum um corpo estranho solto no sistema venoso profundo, próximo ou dentro das câmaras cardíacas. No primeiro caso, trata-se da desconexão entre o cateter como um todo de seu reservatório, e no segundo e terceiro, da fratura do cateter. Tais cateteres são de diferentes fabricantes, mas tinham constituição semelhante, sendo que os dois primeiros eram de polipropileno e o terceiro, de silicone.

As revisões existentes a respeito das complicações relacionadas a cateteres para quimioterapia costumam concordar a respeito da frequência e gravidade das complicações de forma geral, sendo que a ruptura do cateter costuma ocorrer em cerca de 1-4% dos casos^1^^,^ 2 ^,^ 11.

Essa situação pode cursar com trombose local, como no primeiro caso, ou evoluir de forma silenciosa, como no segundo e terceiro casos. Embora nos três casos a evolução tenha sido satisfatória, os eventos provocaram morbidade nesses pacientes, e, de forma geral, essa circunstância traz o risco de determinar arritmias graves, dor precordial e embolização para a artéria pulmonar^12^^-^ 14.

Nos três casos relatados, tanto a desconexão quanto as fraturas ocorreram de forma espontânea. Porém, relatos de desconexão e fragmentação de cateteres podem estar relacionados ao procedimento de retirada^15^. Em estudo realizado por Balsorano, que se destinou especificamente a verificar a integridade dos cateteres retirados, fosse por mau funcionamento ou pelo encerramento do tratamento, evidenciou-se que o tipo do cateter e a utilização de abordagens “heterodoxas” foram relacionados a microrrupturas^16^.

O hospital no qual os CTIs dos casos relatados foram implantados é um centro de referência para tratamento oncológico onde são implantados cerca de 100 CTIs por ano desde 2011. Nesse período, complicações como infecção local, hematoma, trombose no local do cateter e exteriorização do reservatório ocorreram de forma eventual e rara, sem promover grande risco ou morbidade para os pacientes, ainda que não tenhamos os dados precisos a respeito de todos os pacientes.

Até cerca de 90 dias atrás, não tínhamos conhecimento de nenhum caso de fragmentação ou embolização de cateter como os aqui relatados. A população atendida, em boa parte, é moradora de áreas rurais distantes, o que tem dificultado nosso esforço de busca ativa para encontrar esses pacientes e identificar possíveis complicações não relatadas. Soma-se a isso o fato de que parte dos pacientes foi submetida a tratamento com objetivos paliativos e apresentava uma expectativa de vida reduzida.

Nos casos aqui relatados, embora não se possa afirmar claramente as causas, a síndrome de Pinch-off desponta como causa provável para as fraturas, não havendo explicação clara para a desconexão^7^^-^ 9.

O seguimento constante de todos os pacientes em longo prazo poderá levar à elaboração de protocolos que indiquem qual a abordagem e o tipo de cateter mais adequados, a fim de minimizar complicações ou adotar uma profilaxia específica para cada tipo de complicação.

Ainda que as complicações relacionadas à utilização de cateteres para quimioterapia possam ser minimizadas com o estudo individualizado da abordagem mais adequada para cada paciente e a evolução dos materiais, é lógico entender que, a exemplo de outros procedimentos invasivos, o risco sempre estará presente e as complicações se manifestarão eventualmente.
