# Supplementary material for: Desconexão de cateter para quimioterapia: uma complicação rara?
Source: J Vasc Bras. 2016 Oct-Dec;15(4):328–33. [Article in Portuguese] doi: 10.1590/1677-5449.007116 (PMC5829734; doi:10.1590/1677-5449.007116)
Supplement: Translation

# Translation: Detachment of chemotherapy catheters: a rare complication?

**Authors:** Alexandre Faraco de Oliveira, Horácio de Oliveira

**Affiliations:** 1 Universidade do Planalto Catarinense – UNIPLAC, Lages, SC, Brazil.; 2 Clínica Ana Carolina, Lages, SC, Brazil.

**Keywords:** central venous catheters, equipment failure, vascular access devices

## Abstract

Use of totally implantable catheters for chemotherapy treatment is a necessity, but one which brings with it risks of multiple complications, some inherent to insertion of the device and others related to the catheter itself. We describe three cases in which the catheter became disconnected from its reservoir. In the first case, the catheter became completely detached from its reservoir and in the second and third cases the catheter itself underwent fragmentation. In all three cases it was necessary to withdraw the endovascular catheter. This event is described as rare, but it tends to be present in the majority of reviews and it is associated with the risk of serious complications, even though it is often asymptomatic. It is advisable to follow-up patients who have these catheters implanted in order to detect these complications early and to enable understanding of the factors that cause these situations to occur.

## INTRODUCTION

Catheters for infusion of chemotherapy are very useful instruments and are sometimes essential to enable provision of oncological treatment. In many cases, lack of adequate access makes treatment impossible, whether because of irritation of the veins of the upper limbs by the drugs or because of the need for multiple sessions.^1^

Totally implantable catheters (TICs) of the port-a-cath type tend to be the most popular choice because once installed they allow permanent access to a deep vein, which is gained by puncturing the port rather than a vein. In addition to enabling infusion of medications, they can also be used to take blood samples for tests. As the range of chemotherapy treatments available expands and patient survival constantly increases as a result of these treatments, more and more of these catheters are used and for longer periods.^1^

These devices are associated with many complications, related both to placement and to use. The most serious complications, such as pneumothorax or hemothorax, are related to device implantation, tend to manifest immediately, and are the result of choice of puncture site. More common complications, such as hematoma or infection of the puncture site, tend to occur later, but are generally of lower risk to the patient and easily detected.^2^

In this article we describe three cases in which a catheter became detached from its reservoir, with no apparent cause for this event. As a result, the catheter became a foreign body in the deep vein system, specifically the superior vena cava, generating a risk of potentially serious and difficult to diagnose complications. After describing the cases we present a review of existing reports in order to place these complications in relation to them.

## CASE REPORTS

Case 1: a 28-year-old female patient was diagnosed with a left breast tumor during her first gestation and underwent a left mastectomy at approximately 26 weeks. At 34 weeks, a cesarean delivery was performed, followed by placement of a chemotherapy catheter via puncture of the right subclavian vein and placement of the reservoir at the thorax (Figure 1). Around 10 days after implantation of the catheter, the patient complained of right-side cervical pain combined with tumefaction along the path of the right internal jugular vein and localized pain on palpation. A chest X-ray showed that the catheter had become detached from the reservoir (Figure 2). Cervical Doppler ultrasonography showed thrombosis of the internal jugular vein. The patient was put on anticoagulation and exhibited complete regression of the symptoms related to thrombosis after 15 days. Anticoagulation was suspended for 72 hours. Endovascular removal of the catheter was then conducted by catheterization of the right common femoral vein and then anticoagulation was initiated once again.

**Figure 1:** Chest X-ray showing reservoir and catheter implanted via puncture of the right subclavian vein.

**Figure 2:** Catheter detached from the reservoir implanted in the right thorax.

Case 2: a 33-year-old female patient underwent a right mastectomy for right-side breast cancer. Approximately 90 days after surgery, a catheter was implanted for chemotherapy via puncture of the left subclavian vein, with placement of the reservoir in the left thorax. The access was used for five chemotherapy sessions over a 4-month period, during which there were no reports of any problems utilizing the venous access. When she attended for the sixth treatment session, the catheter did not exhibit reflux when punctured, and the patient was referred to the vascular service. A chest X-ray showed that the reservoir and a small segment of the catheter were still implanted in the left thorax, but a piece of the catheter measuring approximately 10 cm was visible on the cardiac projection (Figures 3 4-5). The patient was asymptomatic and reported that she felt no thoracic pain or discomfort, tachycardia, or tachypnea. Endovascular removal of the catheter was then performed via catheterization of the right common femoral vein.

**Figure 3:** X-ray showing the reservoir and part of the catheter implanted in the left thorax, with the greater part of the catheter embolized in the cardiac area.

**Figure 4:** X-ray showing the reservoir and part of the catheter implanted in the left thorax, with the greater part of the catheter embolized in the cardiac area.

**Figure 5:** X-ray showing the reservoir and part of the catheter implanted in the left thorax.

Case 3: a 60-year-old female patient underwent a right mastectomy for right-side breast cancer. Approximately 60 days after surgery, she underwent implantation of a catheter for chemotherapy via puncture of the right subclavian vein, with fixation of the reservoir in the right thorax. The access was used for multiple chemotherapy sessions over a 19-month period, with no reports of problems utilizing the access. When she attended for the twentieth treatment session, the catheter did not exhibit reflux when punctured, and the patient was referred to the vascular service. A chest X-ray showed that the catheter had undergone fragmentation (Figure 6). The patient was asymptomatic and reported that she felt no thoracic pain or discomfort, tachycardia, or tachypnea and was sent for endovascular removal of the catheter.

**Figure 6:** Chest X-ray showing the fragmented catheter and the reservoir in the right thorax.

## DISCUSSION

Implantation of catheters for chemotherapy treatment is a procedure that is already well known and widely used, to the extent that the most frequent complications are also well known.^1^

Some complications are directly related to the puncture site and to implantation of the device. While there are many possibilities for deployment of these catheters, three accesses tend to be used more than others: puncture of the subclavian vein with fixation of the reservoir in the thorax, puncture or dissection of the jugular vein with fixation of the reservoir in the thorax, and dissection of an upper limb vein, the cephalic or the basilic, with fixation of the reservoir in the same upper limb. In all cases, the objective is to place the distal extremity of the catheter in the superior vena cava and to position the reservoir in a manner that facilitates its puncture locally.^3^

The most frequently reported complications, hematoma and infection of the surgical site, are common to all accesses. Insertion of the catheter by dissection, whether of a cervical vein or an upper limb vein, generally results in a longer operating time and dissection of a larger area and sometimes requires sedation combined with local anesthesia.^2^^,^ 4

Access via puncture of the subclavian vein involves risks specific to puncture of deep veins of the thorax, i.e., pneumothorax and hemothorax, and also inadvertent puncture of arteries. However, it is a less traumatic access for the patient and it enables fixation of the reservoir in the superior portion of the thorax, in a site that facilitates puncture. Irrespective of which access is chosen, catheter-related thrombosis is always a possible complication, although it does appear to be more related to access performed by dissection of upper limbs.^2^^,^ 4 ^,^ 5

The risk inherent to puncture related to access via the subclavian vein tends to be minimized by the surgeon’s experience and by using ultrasonography to guide the procedure.^6^ Furthermore, problems related to the puncture itself tend to manifest immediately and are easily identified.^4^

However, access via puncture of the subclavian vein will always involve risk of a specific complication, known as pinch-off syndrome, which is caused by the catheter being subjected to a pincer movement between the clavicle and the first rib, with consequent partial or total fracture of the catheter.^7^^-^ 9 A systematic review of catheters implanted via the subclavian vein demonstrated that damage to the catheter with microruptures may be more common than is realized and that it may be related to the type of material utilized.^10^

Although all TICs have similar shapes, there are specific differences in the choice of materials. The reservoirs may be made of plastic or metal and the catheters themselves are manufactured in silicone or polyurethane. There is evidence that polyurethane catheters are more prone to thrombotic and infectious complications, whereas those made from silicone are more susceptible to mechanical events such as detachment and rupture.^10^

The cases described here involved two different situations that have in common the same result: a foreign body loose in the deep vein system, near to or within the cardiac chambers. In the first case, the entire catheter became disconnected from its reservoir, and in the second and third cases the catheter fractured. The catheters involved were from different manufacturers, but were similarly made, while the first two were polypropylene and the third was silicone.

Published reviews that cover complications related to chemotherapy catheters tend to agree on the frequency and severity of complications in general and show that catheter rupture occurs in around 1-4% of cases.^1^^,^ 2 ^,^ 11

This can progress to local thrombosis, as in the first case described, or may remain silent, as in the second and third cases. Although all three cases had satisfactory outcomes, these events caused the patients morbidity and, in general, such events bring with them the risk of causing serious arrhythmia, precordial pain, and embolization into the pulmonary artery.^12^^-^ 14

In the three cases described, both disconnections and fractures occurred spontaneously. However, there are also reports of detachment and fragmentation of catheters related to removal procedures.^15^ Balsorano conducted a study specifically designed to verify the integrity of catheters after removal, whether because of malfunction or because treatment had been terminated, and found that the type of catheter and use of “heterodox” accesses were related to microruptures.^16^

The hospital in which the TICs were implanted in the cases described here is a referral center for cancer treatment that has implanted around 100 TICs per year since 2011. Over this period, complications such as local infection, hematoma, thrombosis at the catheter site and exteriorization of the catheter reservoir occurred in an occasional and rare manner, without causing serious risk or morbidity to patients, although we do not have precise data for all patients.

Until around 90 days ago, we were unaware of any cases of fragmentation or embolization of catheters like those described here. A significant proportion of the hospital’s population live in remote rural areas, which has created obstacles to our efforts to actively locate these patients and identify possible unreported complications. This factor is compounded by the fact that a proportion of these patients were given treatment with palliative objectives and had limited life expectancies.

In the cases described here, although it is not possible to definitively state the causes, pinch-off syndrome is a probable candidate for the fractures, but there is no obvious explanation for the total detachment.^7^^-^ 9

Constant follow-up of all patients over the long term could lead to development of protocols that indicate which accesses and types of catheter are most appropriate, in order to minimize complications or provide specific prophylaxis for each type of complication.

Although the complications related to use of catheters for chemotherapy can be minimized by conducting individualized studies of the most appropriate access for each patient and by continued development of the materials, it is logical to conclude that, as with other invasive procedures, risk will always be present and complications will manifest from time to time.
